# Laser-evoked cortical responses in freely-moving rats reflect the activation of C-fibre afferent pathways

**DOI:** 10.1016/j.neuroimage.2015.12.042

**Published:** 2016-03

**Authors:** X.L. Xia, W.W. Peng, G.D. Iannetti, L. Hu

**Affiliations:** a Institute of Psychology, Chinese Academy of Sciences, Beijing, China; b Department of Neuroscience, Physiology and Pharmacology, University College London, UK; c Key Laboratory of Cognition and Personality (Ministry of Education), Faculty of Psychology, Southwest University, Chongqing, China

**Keywords:** LEPs, laser-evoked potentials, ECoG, electrocorticography, LMM, linear mixed modeling, FDR, false discovery rate, S1, primary somatosensory cortex, S1FL, the forelimb areas in the S1, S1HL, the hindlimb areas in the S1, S2, secondary somatosensory cortex, ACC, anterior cingulate cortex, Pain, Electrocorticography (ECoG), Animal models, Aδ-fibres, C-fibres, Laser-evoked potentials (LEPs)

## Abstract

The limited success of translating basic animal findings into effective clinical treatments of pain can be partly ascribed to the use of sub-optimal models. Murine models of pain often consist in recording (1) threshold responses (like the tail-flick reflex) elicited by (2) non-nociceptive specific inputs in (3) anaesthetized animals. The direct cortical recording of laser-evoked potentials (LEPs) elicited by stimuli of graded energies in freely-moving rodents avoids these three important pitfalls, and has thus the potential of improving such translation. Murine LEPs are classically reported to consist of two distinct components, reflecting the activity of Aδ- and C-fibre afferent pathways. However, we have recently demonstrated that the so-called “Aδ-LEPs” in fact reflect the activation of the auditory system by laser-generated ultrasounds. Here we used ongoing white noise to avoid the confound represented by the early auditory response, and thereby comprehensively characterized the physiological properties of C-fibre LEPs recorded directly from the exposed surface of the rat brain. Stimulus–response functions indicated that response amplitude is positively related to the stimulus energy, as well as to nocifensive behavioral score. When displayed using average reference, murine LEPs consist of three distinct deflections, whose polarity, order, and topography are surprisingly similar to human LEPs. The scalp topography of the early N1 wave is somatotopically-organized, likely reflecting the activity of the primary somatosensory cortex, while topographies of the later N2 and P2 waves are more centrally distributed. These results indicate that recording LEPs in freely-moving rats is a valid model to improve the translation of animal results to human physiology and pathophysiology.

## Highlights

• We describe the physiological properties of LEPs in freely-moving rats, using ECoG.
• Rat LEPs reflect the activation of C-fibre afferent pathways.
• LEP amplitude reflects stimulus energy and rat nocifensive behavior.
• Similar to their human counterpart, rat LEPs consist of three distinct deflections.
• LEPs in freely-moving rats are a valid model for translational pain research.

## Introduction

Pain is an increasingly important healthcare issue, with dramatic costs for both patient wellbeing and the society (Breivik et al., 2008). Animal models are widely used to understand fundamental mechanisms of chronic pain and identify new analgesic targets. However, the limited success of translating basic findings in animals into effective, clinical analgesics can be largely ascribed to the use of sub-optimal animal models of pain (Mogil, 2009). In this respect, three important limiting factors are (1) the still surprisingly common use of somatosensory stimuli that are neither nociceptive-specific nor quantifiable (e.g., pinching or heating the skin with hot water) (Bastos and Tonussi, 2010, Hernandez et al., 1994, Toda et al., 2008, Uchida et al., 2012), (2) the recording of ‘threshold’ measures (e.g., the tail-flick reflex), instead of suprathreshold responses that allow deriving stimulus–response functions (Carstens and Wilson, 1993, Danneman et al., 1994), and (3) the use of anaesthetized animals when the neural activity of the central nervous system is sampled using electrophysiology or functional magnetic resonance imaging (Ando et al., 2004, Becerra et al., 2011, Toda et al., 2008, Yen and Shaw, 2003). These three important issues can be satisfactorily addressed by combining the selective laser stimulation of skin nociceptors with the recording of the cortical activity using electrodes placed directly on the exposed surface of the brain (electrocorticography, ECoG) in freely-moving rats. Considering that the electrocortical responses elicited by nociceptive stimuli (laser-evoked potentials, LEPs) are also widely used to study pain in healthy individuals and patients (Cruccu et al., 2008, Haanpaa et al., 2011, Treede et al., 2003), the use of similar setups in animal and human studies presents the additional advantage of facilitating successful translation.

Therefore, it is not surprising that laser-evoked cortical responses are being increasingly recorded in animals (Kalliomaki et al., 1993a, Kenshalo et al., 1988, Qiao et al., 2008, Shaw et al., 2001, Tsai et al., 2004). These responses are typically reported as consisting of two distinct components, whose latencies are compatible with the conduction velocity of Aδ-fibres (“Aδ-LEPs”) and C-fibres (“C-LEPs”) (Isseroff et al., 1982, Qiao et al., 2008, Shaw et al., 2001). However, we have recently demonstrated that the so-called “Aδ-LEPs”, instead of reflecting the activation of the Aδ-nociceptive system (Hu et al., 2015), is actually consequent to the activation of the auditory system by laser-generated ultrasounds that can be detected by rats, but not by humans (Panksepp and Burgdorf, 2003, Scruby and Drain, 1990). This auditory response has been so far mistakenly interpreted as reflecting the Aδ-somatosensory input, thus undermining the conclusions of several previous investigations (Isseroff et al., 1982, Qiao et al., 2008, Shaw et al., 2001). Important from a practical perspective, this auditory response can be effectively eliminated by delivering laser pulses during ongoing auditory white noise (Hu et al., 2015).

Here, we delivered nociceptive-specific laser pulses to 12 awake, freely-moving rats. We recorded their behavioral and neurophysiological responses using direct recording of the electrical activity of the cerebral cortex, avoiding the confound represented by the laser-induced early auditory response. We aimed to test (1) whether reliable LEP responses can be obtained in single animals; (2) which population of peripheral nociceptors is reflected in the LEP responses; (3) the dependency of LEP responses on the stimulated territory (i.e., forepaws and hindpaws on the right and left sides); (4) the dependency of LEP responses on stimulus energy, and their relation with nocifensive behavior. Finally, we propose an optimal montage to isolate different LEP components arising from different neural generators.

## Methods

### Animal preparation and surgical procedures

We used 12 adult male Sprague–Dawley rats weighing between 300 and 400 g. Rats were housed in cages under temperature- and humidity-controlled conditions. All rats received food and water ad libitum, and were kept in a 12-h day–night cycle (lights on from 08:00 to 20:00). All surgical and experimental procedures were approved by the ethics committee of Southwest University.

Prior to the surgery, rats were anesthetized with sodium pentobarbital (50 mg/kg, intraperitoneal injection: i.p.). Supplementary doses (12.5 mg/kg, i.p.) of sodium pentobarbital were given to maintain appropriate anesthetic depth during surgery, when necessary. During anesthesia the rat head was fixed using a stereotaxic apparatus. After the dorsal aspect of the scalp was shaved, the skull was exposed by a midline incision, as previously described (Qiao et al., 2008, Shaw et al., 1999, Shaw et al., 2001). Fourteen holes were drilled on the skull, at defined locations on the stereotaxic reference system (Shaw et al., 1999). Stainless steel screws (diameter = 1 mm) were inserted into the holes, without penetrating the underlying dura mater. Twelve screws acted as active electrodes, and their coordinates in respect to the bregma were as follows (in mm; positive X and Y axis values indicate right and anterior locations, respectively). FL1: X = − 1.5, Y = 4.5; FR1: X = 1.5, Y = 4.5; FL2: X = − 1.5, Y = 1.5; FR2: X = 1.5, Y = 1.5; LFL: X = − 4.5, Y = 0; RFR: X = 4.5, Y = 0; PL1: X = − 1.5, Y = − 1.5; PR1: X = 1.5, Y = − 1.5; LPL: X = − 4.5, Y = − 3; RPR: X = 4.5, Y = − 3; PL2: X = − 1.5, Y = − 4.5; PR2: X = 1.5, Y = − 4.5. The reference and ground electrodes were placed on the midline, 2 mm and 4 mm caudally to the Lambda, respectively. The wires coming from each electrode were held together with a connector module fixed on the scalp with dental cement. To prevent post-surgical infections, rats were injected with penicillin (60,000 U, i.p.) immediately after the surgery. Following the surgery, rats were kept in individual cages for at least 7 days before the LEP experiments.

### Experimental protocol

Radiant-heat stimuli were generated by an infrared neodymium yttrium aluminum perovskite (Nd:YAP) laser with a wavelength of 1.34 μm (Electronical Engineering, Italy). Nd:YAP laser pulses activate directly cutaneous nociceptive terminals in the most superficial skin layers (Baumgartner et al., 2005, Iannetti et al., 2006, Sikandar et al., 2013). The laser beam was transmitted via an optic fibre and its diameter was set at approximately 4 mm (~ 13 mm^2^) by focusing lenses. A He–Ne laser pointed to the stimulated area. Laser pulses were delivered to four body territories (left forepaw, right forepaw, left hindpaw, and right hindpaw), using five stimulus energies (E1–E5: 1–4 J in steps of 0.75 J). The pulse duration was 4 ms, and the interval between two consecutive stimuli was never shorter than 30 s. To avoid nociceptor fatigue or sensitization, the target of the laser beam was displaced after each stimulus (Leandri et al., 2006).

During ECoG data collection, rats were placed into a plastic cage (30 × 30 × 40 cm^3^), whose floor had a regular series of holes through which the laser beam could pass and reach the animal's skin (Hu et al., 2015). The diameter of each hole was 5 mm, and the distance between the borders of two nearby holes was 2 mm. The cage ceiling had a single, larger hole (diameter = 15 cm) through which ECoG cables were connected to the amplifier. Before the ECoG experiment, rats were placed for at least four slots of 1 h each in the same plastic cage, to familiarize them with the recording environment. In both pre-recording and recording sessions, rats could freely move in the cage. The skin area targeted by the laser was always within the paw. It was defined by the region of the paw that was visible through the holes in the bottom side of the cage, when the rat was spontaneously still. The distance between the laser end piece and the target site was kept constant at ~ 1 cm.

As demonstrated in our previous study (Hu et al., 2015), laser stimulation of the skin generates ultrasounds detected by the rat auditory system (Moller, 2013, Panksepp and Burgdorf, 2003, Scruby and Drain, 1990, Zhang, 1992). This has been further tested in the present study, by recording the thermoelastic response elicited by the laser stimulation of the plastic material of the cage surrounding the animal using a tunable ultrasound detector (Mini-2 Bat Detector, SUMMIT, Birmingham, UK). This recording showed a clear response in the ultrasound range (~ 40–60 kHz), graded with the energy of the laser pulse (Supplementary Fig. 1 and Supplementary audio files). Therefore, to avoid the activation of the auditory system by the laser-generated ultrasounds, the ECoG recording was performed during ongoing white noise, a procedure that allows selective recording of LEPs related to the activation of the nociceptive system. We delivered 10 laser pulses at each of the five stimulus energies (E1–E5) and each of the four stimulation sites (left forepaw, right forepaw, left hindpaw, and right hindpaw), for a total of 200 pulses. The order of stimulated sites was pseudorandomized. Animals were video-recorded throughout the experiment, and nocifensive behavioral scores were assigned after each laser stimulus, according to previously-defined criteria based on the animal movement (Fan et al., 2009, Fan et al., 1995), as follows: no movement (score = 0), head turning (including shaking or elevating the head; score = 1), flinching (i.e., a small abrupt body jerking movement; score = 2), withdrawal (i.e., paw retraction from the laser stimulus; score = 3), licking and whole body movement (score = 4). The effect of stimulus energy and stimulation site on behavioral scores was assessed using a two-way repeated-measures analysis of variance (ANOVA), with ‘stimulus energy’ (five levels: E1–E5) and ‘stimulation site’ (four levels: left forepaw, right forepaw, left hindpaw, and right hindpaw) as within-subject factors.

### ECoG recording and data analysis

#### Data preprocessing

ECoG data were recorded with a sampling rate of 1000 Hz (Brain Products), and preprocessed using EEGLAB (Delorme and Makeig, 2004), an open source toolbox running in the MATLAB environment. Continuous ECoG data were band-pass filtered between 1 and 30 Hz. ECoG epochs were extracted using a window analysis time of 1500 ms (− 500 ms to + 1000 ms with respect to the stimulus), and baseline corrected using the pre-stimulus interval.

Average LEP waveforms time-locked to the onset of the laser stimulus were computed for each animal and experimental condition. Single-animal average waveforms were subsequently averaged to obtain group-level waveforms. Group-level scalp topographies were computed by spline interpolation. The boundary of the scalp topography was determined based on the stereotaxic atlas (Paxinos and Watson, 2006).

#### Linear mixed modeling (LMM)

To explore the trial-by-trial relationship between LEP amplitude and either stimulus intensity or nocifensive behavioral score, we applied a linear mixed model (LMM) to the data (McCulloch et al., 2008, Verbeke and Molenberghs, 2009). By taking into account the correlation of these measures at within-subject level, the LMM estimates the intraindividual dependence of LEP amplitude on stimulus intensity (or nocifensive behavioral score). Thus, stimulus intensities (or nocifensive behavioral scores) were used as independent variables (responders), and LEP amplitudes at each electrode and time point were used as dependent variable (predictor) (Schulz et al., 2011). This analysis results in a time-varying statistical *t* value for each electrode and time point, which reflected the strength of relationship between LEP amplitude and stimulus intensity (or nocifensive behavioral score). To account for multiple comparisons, the significance level (p value) was corrected using a false discovery rate (FDR) procedure (Benjamini and Hochberg, 1995).

#### ECoG montages

To describe scalp distributions of different ECoG components in the time domain (i.e., N1, N2, and P2 waves), LEP waveforms were re-referenced to a common average reference. Since the N1 wave had a lateralized topography compatible with the somatotopical organization of the contralateral primary somatosensory cortex (S1), its latency and amplitude were measured at the following electrodes: RFR (left forepaw), LFL (right forepaw), PR1 (left hindpaw), and PL1 (right hindpaw). Since the N2 and P2 waves were maximal at central and frontal regions respectively, their latencies and amplitudes were measured from PL1 + PR1 and FL1 + FR1 electrodes, respectively. To demonstrate the validity of these montages for detecting isolated LEP components, we showed the spatial correspondence between the scalp location of the electrode pair defining each montage and the possible neural generators of the recorded signal. The locations of the forelimb and hindlimb areas in the S1 (S1FL and S1HL), of the secondary somatosensory cortex (S2), the insula, and the anterior cingulate cortex (ACC), were derived from the 3D Paxinos and Watson atlas of the rat brain (Hjornevik et al., 2007, Paxinos and Watson, 2006), and overlaid on the 3D brain mask surface from the Waxholm Space atlas of the rat brain (Arganda-Carreras et al., 2006, Papp et al., 2014).

## Results

### Nocifensive behavioral scores

**Fig. 1:**
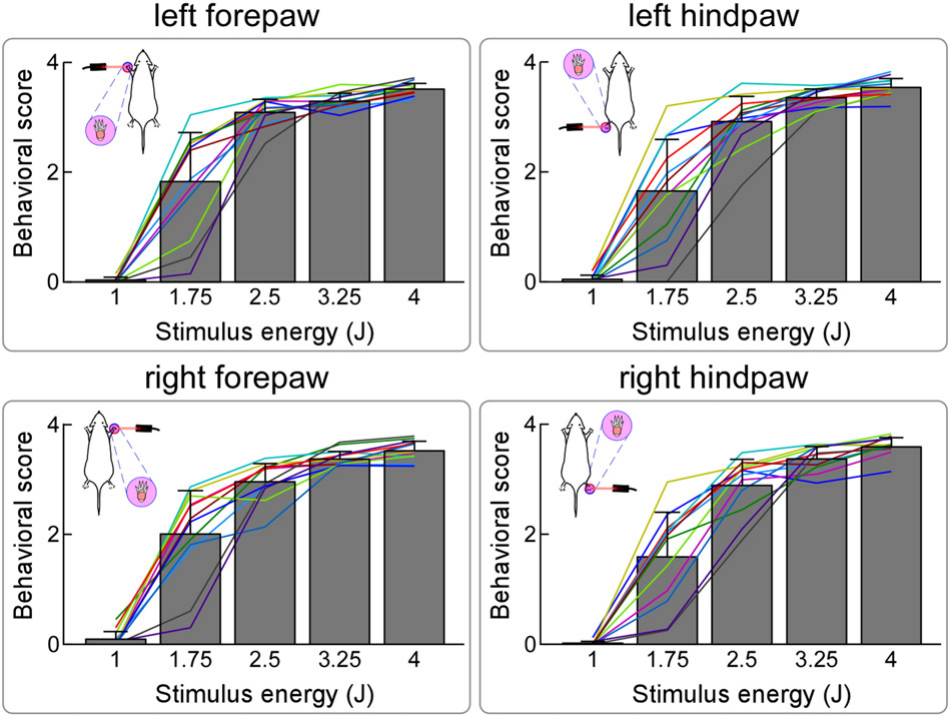
Effect of stimulus energy and stimulation site on nocifensive behaviors. Stimulus-induced nocifensive behaviors were quantified as follows: no movement (score = 0), head turning (including shaking or elevating the head; score = 1), flinching (i.e., a small abrupt body jerking movement; score = 2), withdrawal (i.e., paw retraction from the laser stimulus; score = 3), licking and whole body movement (score = 4). Behavioral scores are significantly modulated by stimulus energy (F = 161.2, p < 0.001), but not by stimulation site (F = 1.94, p = 0.14). Colored lines represent single-animal behavioral scores.

Nocifensive behavioral scores elicited by graded laser stimuli delivered to different body sites are summarized in Fig. 1, and their proportional values are reported in Table 1. Two-way repeated-measures ANOVA revealed that behavioral scores were significantly modulated by stimulus energy (F = 161.2, p < 0.001), but not by stimulation site (F = 1.94, p = 0.14). Post-hoc pairwise comparisons revealed that behavioral scores were significantly larger when stimulus energies were stronger (p < 0.05 for all comparisons, Bonferroni corrected).

### Brain responses: waveforms and scalp topographies

**Fig. 2:**
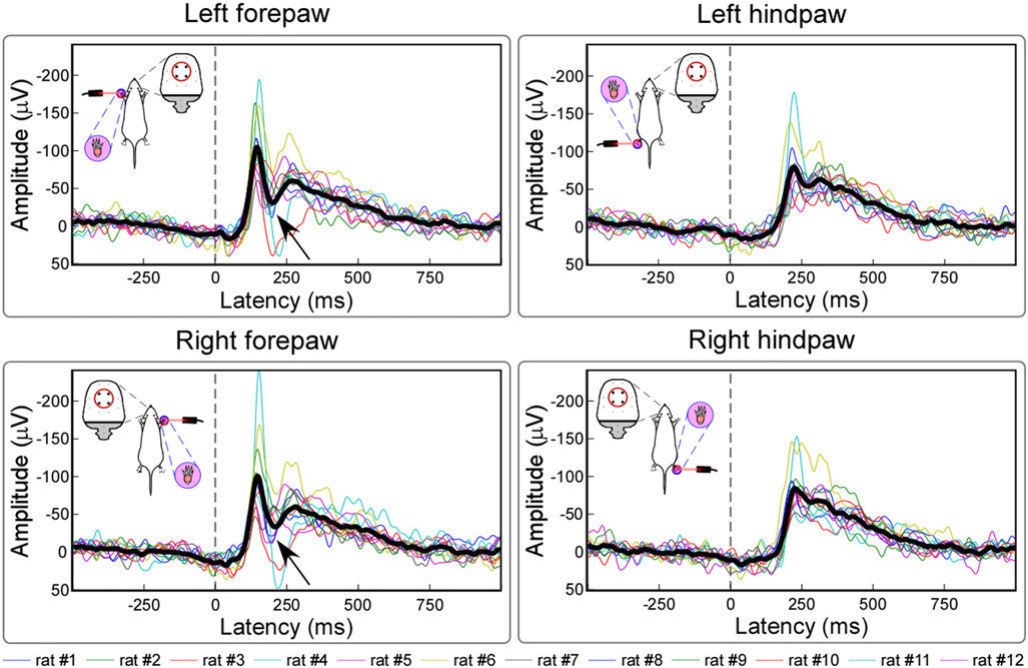
Single-animal and group-level LEP waveforms. Displayed signals were recorded from central electrodes (FL2, FR2, PL1, and PR1), using the electrode located 2 mm caudal to the Lambda as reference. Colored waveforms represent single-animal averages, whereas the black waveform is the group-level average.

**Fig. 3:**
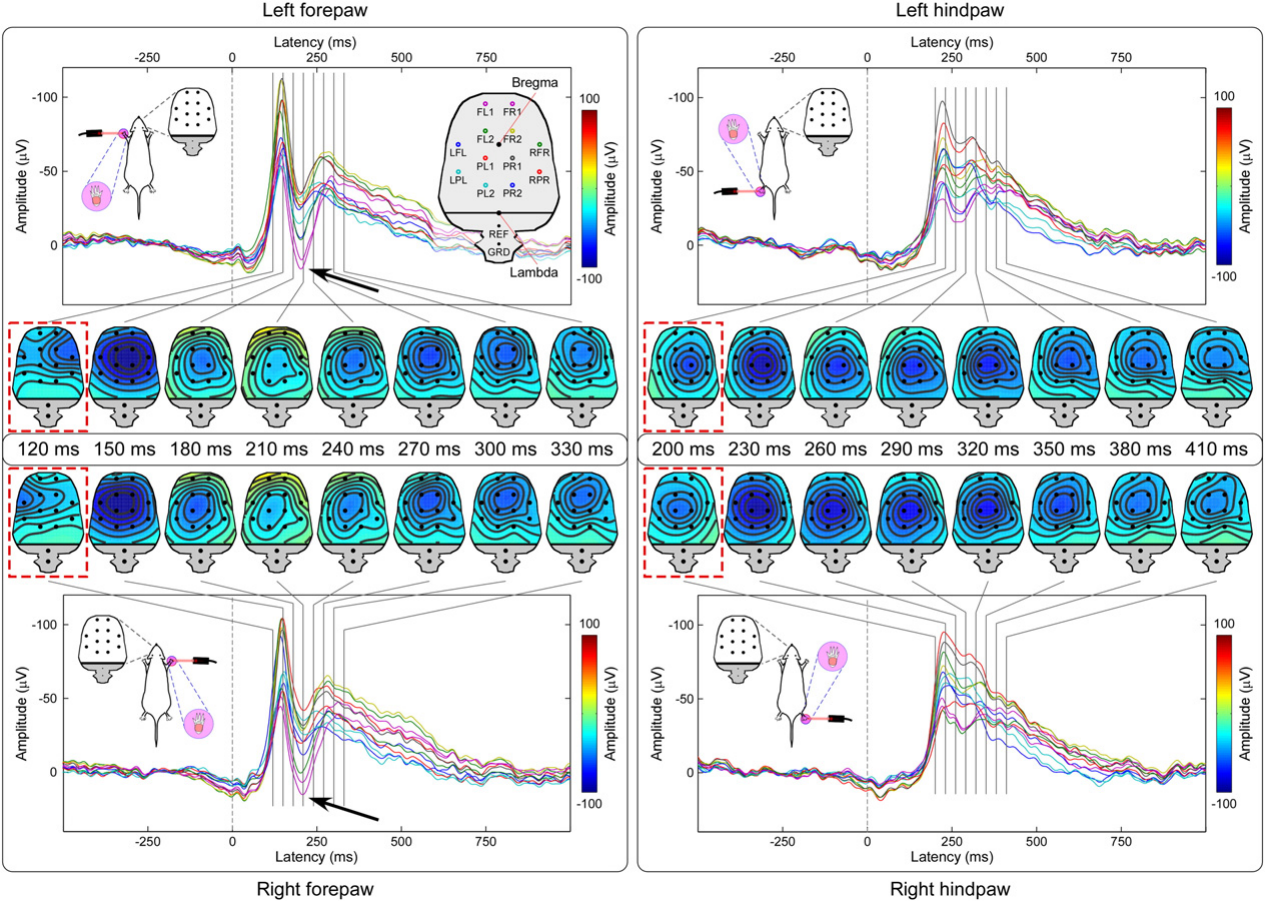
Group-level LEP waveforms and scalp topographies. Signals from different electrodes are plotted in different colors and superimposed. The positions of the 14 ECoG electrodes are displayed in the top left of each plot. Scalp topographies are displayed at the intervals 120–330 ms (forepaw stimulation, left panels) or 200–410 ms (hindpaw stimulation, right panels). The scalp topography of the early part of forepaw response (120 ms, highlighted in red) displays a negativity contralateral to the stimulated side, while that of the hindpaw response (200 ms, highlighted in red) is centrally distributed and not different between stimulated sides. In contrast, the scalp topographies of the middle and late parts of the LEP waveform are strikingly similar in both the forepaw and hindpaw responses.

Laser pulses elicited a clear LEP response in all animals. Fig. 2 shows the average waveforms of individual animals and the group-level average waveform, from the four central electrodes (FL2, PL1, FR2, and PR1). Fig. 3 shows the group-level average waveforms from each of the 12 electrodes, together with the scalp distribution of the signal in the time window 120–330 ms (forepaw stimulation) or 200–410 ms (hindpaw stimulation).

When signals were displayed using the electrode located 2 mm caudal to the Lambda as reference, the response consisted of a brisk negative deflection, followed by a slower increase of amplitude that went back to baseline after approximately 800 ms (Fig. 2, Fig. 3). The latency and amplitude of the main negative deflection were as follows (mean ± SEM; the same hereinafter). Left forepaw: 146 ± 3 ms, − 111.7 ± 12.3 μV; right forepaw: 146 ± 2 ms, − 106.3 ± 16.2 μV; left hindpaw: 229 ± 4 ms, − 91.3 ± 9.1 μV; and right hindpaw: 225 ± 5 ms, − 86.7 ± 11.3 μV. The conduction velocity of peripheral afferents mediating the LEP response, estimated on the basis of the latency difference between the responses elicited by forepaw and hindpaw stimulation, was 0.89 ± 0.13 m/s. This figure is clearly compatible with the physiological properties of C-fibre afferents (Shaw et al., 1999).

Embedded in the long-lasting negative deflection, there was also a small and transient increase of amplitude (marked with arrows in Fig. 2, Fig. 3), which was only clear in the LEPs elicited by the forepaw stimulation. The latency and amplitude of this transient amplitude increase were as follows. Left forepaw: 197 ± 5 ms, − 20.6 ± 9.7 μV; right forepaw: 203 ± 6 ms, − 25.5 ± 10.3 μV.

The scalp topography of the earliest part of the response elicited by forepaw stimulation (120 ms) displayed a clear negative maximum on the hemisphere contralateral to the stimulated side. In contrast, the scalp topography of the earliest part of the response elicited by hindpaw stimulation (200 ms) was more centrally distributed. This observation is strongly evocative of a somatotopical organization, suggesting that the earliest part of the LEP response likely reflects the activity of the primary somatosensory cortex.

In contrast, the scalp topography of the main negative peak was remarkably similar across the four stimulated territories, with a maximum around central electrodes (approximate latency: 150 ms following forepaw stimulation; 230 ms following hindpaw stimulation). The scalp topography of the following transient positive deflection (approximate latency: 210 ms, only detectable following forepaw stimulation) within the ascending branch of the main negative wave was also similar following right and left forepaw stimulation, and had a relative positive maximum centered on frontal electrodes (FL1 and FR1).

### Stimulus–response and behavior–response functions

**Fig. 4:**
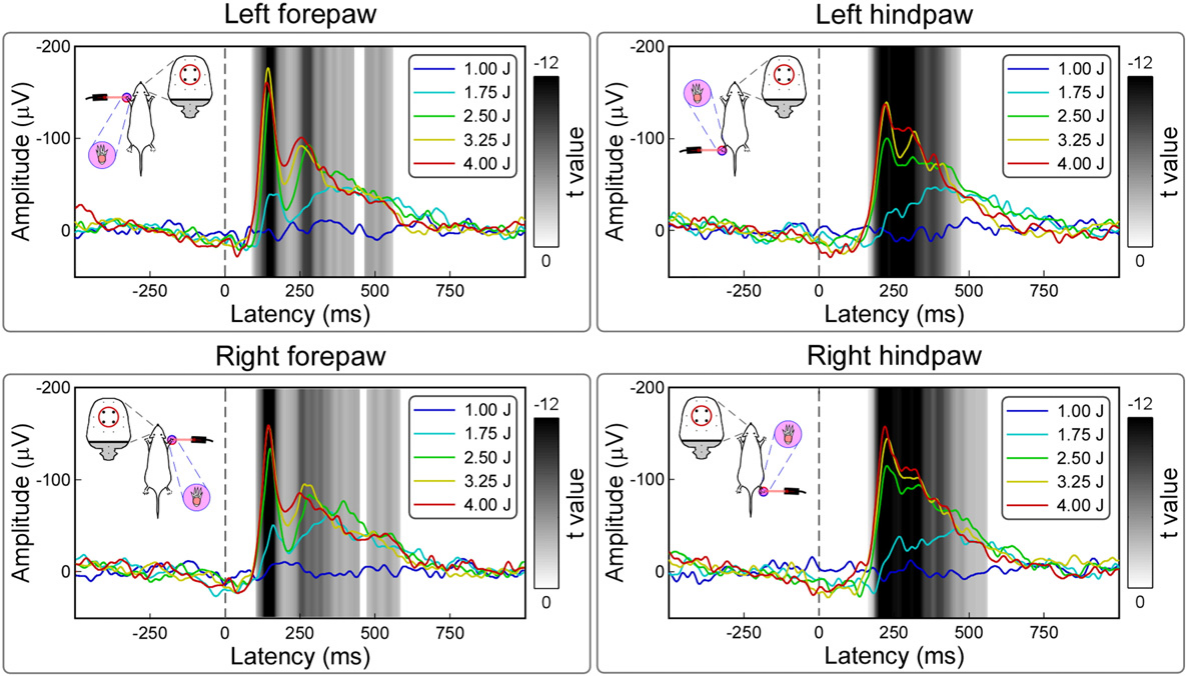
Point-by-point effect of stimulus energy on LEP amplitude. Significant relationship between stimulus energy (E1–E5) and LEP amplitude is coded in gray (*t* values obtained by linear mixed model; p values are FDR corrected).

The amplitude of the LEP response displayed a clear monotonic relationship with stimulus energy (Fig. 4). LMM analysis revealed that, for each stimulation site, the signal was significantly larger when higher energy of stimulation was delivered. This energy-dependent effect was significant in large time intervals of the responses (p_fdr_ < 0.05). For the four central electrodes (FL2, PL1, FR2, and PR1), these intervals were as follows: 93–435 ms and 461–553 ms (left forepaw); 101–451 ms and 483–567 ms (right forepaw); 173–462 ms (left hindpaw); 174–537 ms (right hindpaw).

**Fig. 5:**
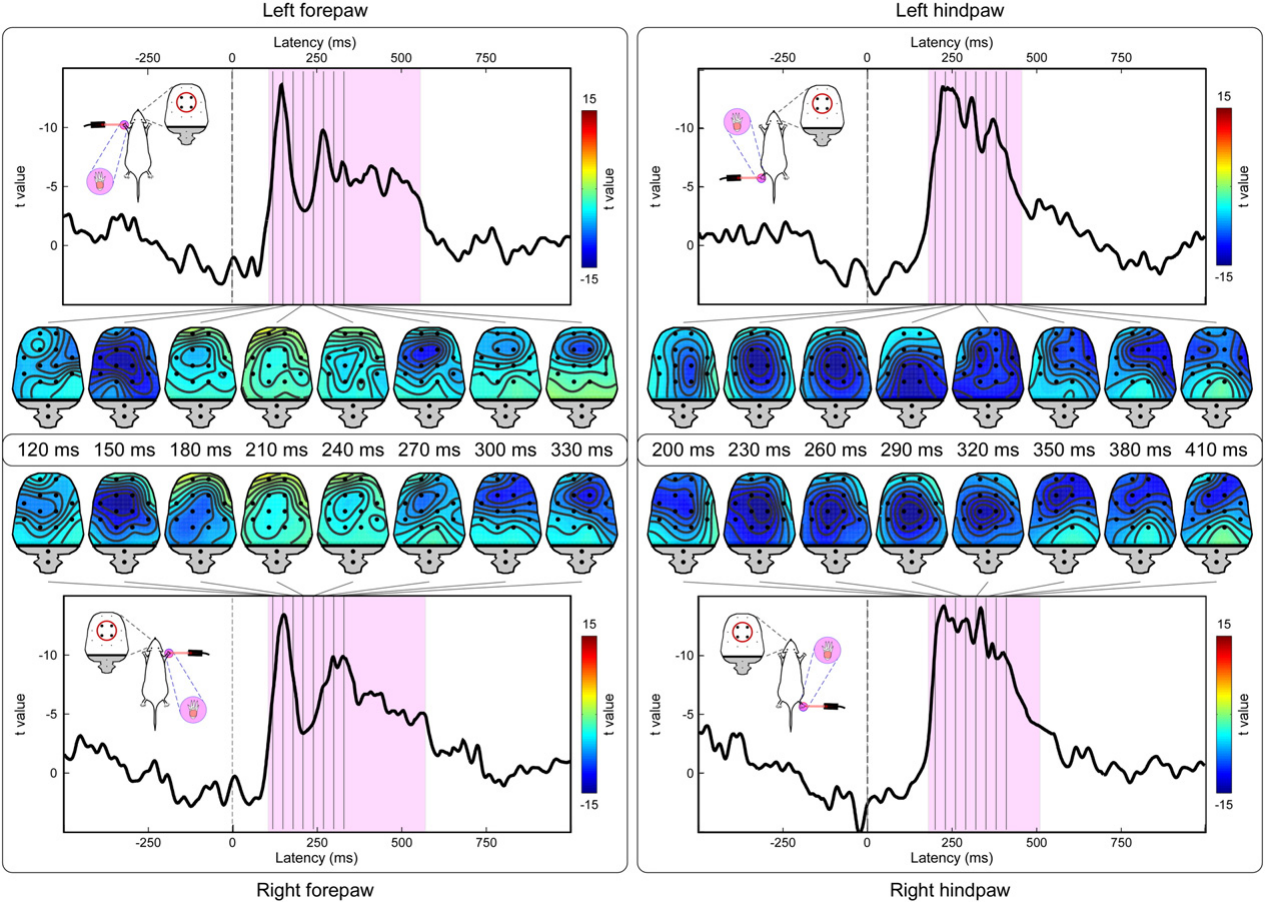
Point-by-point relationship between LEP amplitudes and nocifensive behavioral scores. Waveforms represent the time course and scalp topographies of *t* values for different stimulation sites (top left: left forepaw; bottom left: right forepaw; top right: left hindpaw; bottom right: right hindpaw). *t* values, obtained by linear mixed model, reflect the strength of the relationship between behavioral scores and signal amplitudes. Significant time intervals are highlighted in pink (p values are FDR corrected).

The amplitude of the LEP response was also significantly related to nocifensive behavioral score. Fig. 5 shows the result of the LMM analysis, with the time courses of the *t* value expressing the significance of the relationship between signal amplitude and nocifensive score at each time point. This relationship was significant almost throughout the entire LEP response. For the four central electrodes (FL2, PL1, FR2, and PR1), this significant relationship occurred in the following intervals: 104–565 ms (left forepaw); 103–582 ms (right forepaw); 177–479 ms (left hindpaw); 178–550 ms (right hindpaw) (p_fdr_ < 0.05). The scalp distribution of these *t* values was similar to that of the absolute LEP amplitudes, indicating that virtually the entire ECoG response amplitude was related to nocifensive behavioural scores. For example, the scalp topography of the early part of the *t*-value time course expressing the relationship between amplitude of the LEP elicited by forepaw stimulation and nocifensive scores (120 ms) displayed a clear maximum on the hemisphere contralateral to the stimulated side, while the scalp topography of the early part of the *t*-value time course expressing the relationship between amplitude of the LEP elicited by hindpaw stimulation and nocifensive scores (200 ms) was more centrally distributed.

### Optimal montages to isolate LEP components

Based on the scalp distribution of the LEP response, we defined some ECoG montages that allow isolating the different LEP components as separate peaks in the time domain. To eliminate the influence of activities widespread across the scalp, we displayed the data using an average reference, a procedure that removes the global background activity and thus enhances spatially-discrete activities (Bertrand et al., 1985). When examining the average-reference data, three clear deflections could be identified: a first negative wave (N1) followed by a second negative (N2) and a third positive wave (P2).

**Fig. 6:**
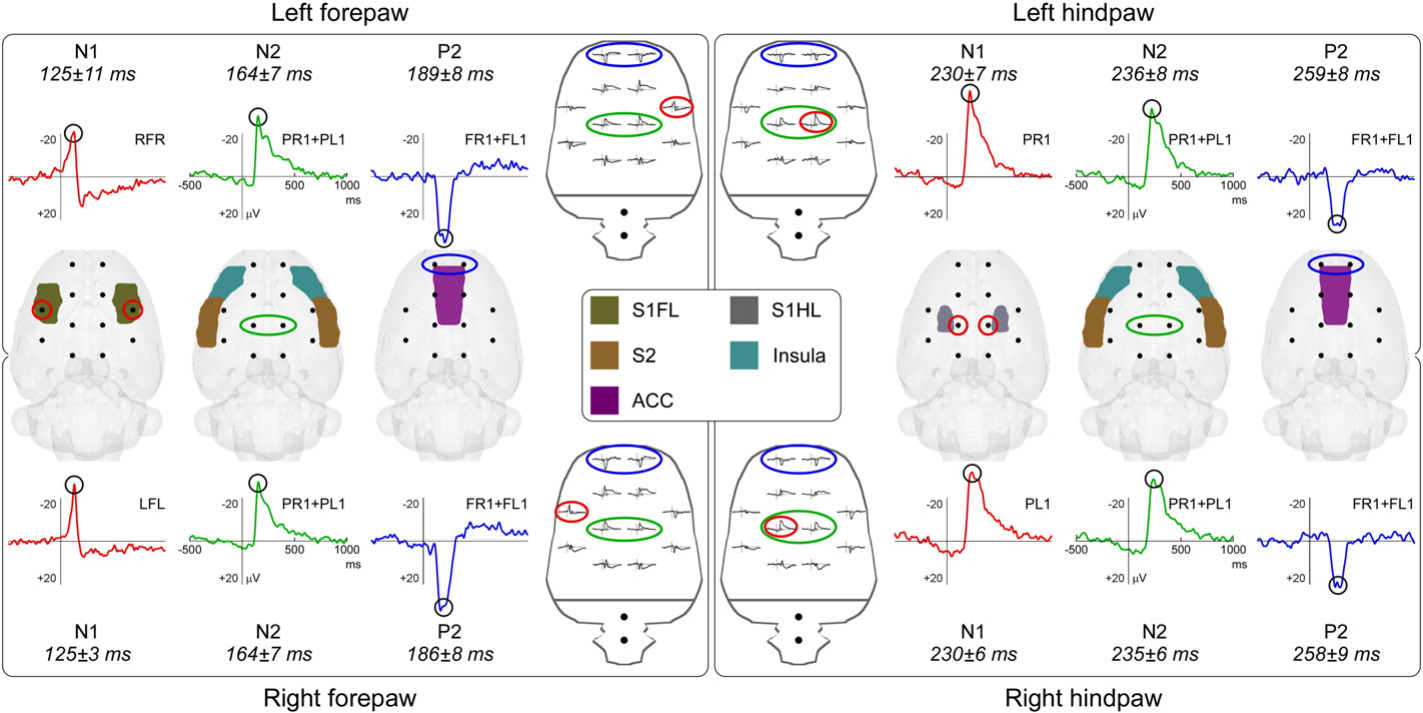
Principal LEP waves (N1, N2, and P2) and their possible underlying anatomical structures. The early-latency N1 wave (red waveforms) shows a maximum on the electrodes (red circles) overlying the relevant areas of the primary somatosensory cortex. The N2 wave (green waveforms) is maximal at central electrodes (green circles), and possibly reflects the combined activity of bilateral S2 and insula. The P2 wave (blue waveforms) is maximal at frontal electrodes (blue circles), and possibly reflects the activity of the anterior cingulate cortex.

The N1 wave was optimally detected from electrode RFR (following left forepaw stimulation), LFL (following right forepaw stimulation), PR1 (following left hindpaw stimulation), and PL1 (following right hindpaw stimulation) (Fig. 6). This observation confirms that the scalp distribution of this component is somatotopically organized, and is suggestive of an underlying source located in the primary somatosensory cortex contralateral to the stimulated paw. The peak latency and amplitude of the N1 wave were as follows: 125 ± 11 ms, − 35.7 ± 6.2 μV (left forepaw); 125 ± 3 ms, − 29.3 ± 6.0 μV (right forepaw); 230 ± 7 ms, − 42.0 ± 6.5 μV (left hindpaw); 230 ± 6 ms, − 34.3 ± 5.4 μV (right hindpaw).

The N2 wave was optimally detected from central electrodes (PL1 and PR1), regardless of stimulated district (Fig. 6). The centrally distributed topography of this peak makes the inference of its underlying source less straightforward. One possibility is that the N2 wave is generated in the S2 and insula, bilaterally. The peak latency and amplitude of the N2 wave were as follows: 164 ± 7 ms, − 31.2 ± 2.8 μV (left forepaw); 164 ± 7 ms, − 30.3 ± 3.0 μV (right forepaw); 236 ± 8 ms, − 34.0 ± 4.4 μV (left hindpaw); 235 ± 6 ms, − 31.1 ± 4.2 μV (right hindpaw).

Finally, the P2 wave was optimally detected from frontal electrodes (FL1 and FR1), regardless of the stimulated district (Fig. 6). The frontal symmetrical topography is compatible with an underlying generator in the ACC. The peak latency and amplitude of the P2 wave were as follows: 189 ± 8 ms, 38.5 ± 6.1 μV (left forepaw); 186 ± 8 ms, 41.9 ± 5.0 μV (right forepaw); 259 ± 8 ms, 29.8 ± 5.1 μV (left hindpaw); 258 ± 9 ms, 26.6 ± 6.7 μV (right hindpaw) (Fig. 6).

## Discussion

In this study we provide a full description of the ECoG responses elicited by selective laser stimulation of nociceptive afferents in freely-moving rats. All experiments were conducted while white noise was continuously played throughout the recording session. Indeed, we recently showed that the early part of the cortical response elicited by laser stimulation reflects the ultrasound-induced activation of the auditory system and not, as previously thought, of the Aδ-nociceptive pathways (Hu et al., 2015). The ongoing white noise masks the laser-induced ultrasound and therefore abolishes the auditory-evoked transient response (Hu et al., 2015).

We obtained four main results. First, we showed that a clear LEP response can be recorded in single animals, following the stimulation of each of the four paws (Fig. 2). Second, this LEP response reflects the activation of C-fibre afferent pathways. Third, the LEP response amplitude is positively related to the energy of the noxious stimulus, and also reflects the score of stimulus-evoked nocifensive behaviors (Fig. 4, Fig. 5). Fourth, we identified different LEP peaks with distinct topographies, probably reflecting different sets of underlying sources. While the scalp topography of the early components (N1 wave) is somatotopically organized, and likely reflects the activity of the primary somatosensory cortex, the scalp topography of later components (N2 and P2 waves) is more likely to reflect the activity of bilateral generators in the S2, insula, and ACC (Fig. 3, Fig. 6).

Finally, we propose optimal ECoG montages to isolate LEP peaks (N1, N2, and P2 waves) that reflect functionally-distinct neural activities (Fig. 6).

These functional properties are reminiscent of those of the LEPs recorded in healthy human participants. This similarity indicates that recording LEPs in freely-moving rats is a valid model to translate experimental animal results into human physiology and pathophysiology.

### Rat LEPs reflect C-fibre activation

We showed that laser-evoked brain potentials can be easily identified in the ECoG of individual freely-moving rats (Fig. 2). Based on the latency difference of the cortical response elicited by forepaw and hindpaw stimulation, we demonstrated that the afferent somatosensory input eliciting the cortical response reflects the activation of C-fibre nociceptive pathways (Fig. 2, Fig. 3). Indeed, several studies recording LEPs in freely moving rats (Isseroff et al., 1982, Qiao et al., 2008, Shaw et al., 1999, Shaw et al., 2001) have described clear responses at latencies compatible with the conduction velocity of both Aδ-fibres (“Aδ-LEPs”) and C-fibres (“C-LEPs”), a finding also consistent with human studies (Hu et al., 2014). However, when LEPs were recorded in halothane-anesthetized rats (Kalliomaki et al., 1998, Kalliomaki et al., 1993a, Kalliomaki et al., 1993b, Schouenborg et al., 1986), the results were similar to what we describe in the present study (Fig. 3), i.e., only components with latency compatible to the activation of C fibres could be identified. The fact that only C-LEPs were present in these studies was explained by the lower resistance of “Aδ-LEPs” to the effect of anesthesia (Shaw et al., 1999).

However, we have recently provided compelling evidence that the so-called “Aδ-LEPs” simply reflects the activation of the murine auditory system by the ultrasounds generated by the laser stimulation of the skin through a well-known thermo-elastic mechanism (Hu et al., 2015). This ultrasound, which can be detected by rats but not by humans, elicits a clear auditory response that has been misinterpreted as reflecting the activation of the Aδ-nociceptive pathways. Our recent results undermine the interpretation that the cortical response elicited by nociceptive-specific laser pulses in rats reflects the activation of both Aδ and C pathways (Isseroff et al., 1982, Qiao et al., 2008, Shaw et al., 2001). Indeed, the lack of “Aδ-LEPs” during anesthesia can be parsimoniously explained by the well-known observation that, compared with somatosensory-evoked brain responses, auditory ERPs are more strongly affected by halothane and halothane–fentanyl anesthesia (Kochs et al., 1990). For this reason, all experiments performed in the present study have been conducted using ongoing auditory white noise, a procedure that effectively avoids the early auditory-related response (Hu et al., 2015).

The present data clearly indicate that ECoG responses recorded in freely-moving rats only reflect neural activities elicited by the arrival of the C-fibre input to the central nervous system. This finding is not surprising, as primate and rodent peripheral nociceptors have very different sensitivities to noxious heat stimulation (Lynn and Shakhanbeh, 1988, Shim et al., 2005): while Aδ-nociceptors are highly sensitive to heat in primates (Magerl et al., 1999, Treede et al., 1998), they are virtually insensitive to heat in rodents (Lynn and Shakhanbeh, 1988, Shim et al., 2005). In contrast, both primate and rodent C nociceptors show graded responses to heat stimuli of different intensities (Darian-Smith et al., 1979, Hu et al., 2014, Kalliomaki et al., 1993b, Sikandar et al., 2013). The graded activation of peripheral C-nociceptors in response to different energies of laser stimuli is likely to represent the physiological basis of the stimulus–response functions we observed at cortical level (Fig. 4).

Still, we cannot completely exclude the possibility that nocifensive movements contributed to the LEP recordings. However, in line with previous studies (Kalliomaki et al., 1993a, Shaw et al., 1999, Shaw et al., 2001) C-LEPs recorded on rats were cortical in origin for the following reasons. First, C-LEPs could be clearly recorded, even in trials without nocifensive movement (see also Fig. 5 in Shaw et al., 2001). Second, the scalp topographies of C-LEP responses were clearly different from those one would expect as consequence of activity in distant muscles (Fig. 6). Nevertheless, despite these suggestions that the recorded signal was cortical in origin, we are not able to rule out the possibility that the brain responses were to some extent contaminated by nocifensive related movements.

### C-LEPs in freely-moving rats: response features and functional significance

We have characterized the stimulus–response functions of C-LEPs in freely-moving rats: the LEP response amplitude is positively related to the energy of the eliciting stimulus, as well as to the scores of nocifensive behaviors (Fig. 4, Fig. 5). When displayed using an average reference, C-LEPs consist of three distinct peaks, whose polarity, order and topography are surprisingly similar to the same features of human LEPs (Fig. 6). The observation that the earliest part of the C-LEPs evoked by forepaw stimulation was maximal contralaterally to the stimulated side, while that of the C-LEPs evoked by hindpaw stimulation was more centrally distributed (Fig. 3) is important, as it indicates that S1 is the first cortical region activated by the afferent spinothalamic input, even if only unmyelinated (Fig. 6). This finding suggests that S1 could also contribute to the early part of C-LEPs in humans, which, to the best of our knowledge, is still unknown. In contrast, later parts of the C-LEP response are more likely to reflect the activity of the S2, insula, and the ACC, bilaterally (Fig. 3, Fig. 6).

Therefore, all the basic properties of the murine C-LEP response match well with what is observed in human LEP recordings (Opsommer et al., 2001), although it should not be forgotten that the first LEP response in humans reflects the activation of the Aδ afferent pathways (Hu et al., 2014). This strong similarity between murine and human LEPs has two important implications.

First, from a pragmatic perspective, it indicates that recording C-LEPs in freely-moving rodents is a viable way to achieve a successful translation of experimental results from rodents to humans. This will allow the exploration of the function of C-fibre nociceptive pathways in murine models of clinically-relevant painful conditions, including neuropathic pain (Mogil, 2009, Mogil et al., 2010), as well as a better translation of rodent pharmacological findings to humans. The latter point is particularly important, given the limited success of translating basic findings into effective clinical analgesics, because of the use of sub-optimal animal models (Mogil, 2009). The technique described in this paper represents a clear improvement over previous animal models of nociceptive function.

Second, from a neuroscientific perspective, it suggests that the brain responses elicited by transient nociceptive stimuli have a similar functional significance in rodents and humans. Therefore, it is likely that, as repeatedly demonstrated in human LEPs, the largest part of murine C-LEPs do not reflect nociceptive-specific neural processing. Instead, they might reflect multimodal neural activities possibly related to the detection and reaction to behaviourally-relevant stimuli in the sensory environment (Moayedi et al., 2015, Mouraux and Iannetti, 2009, Mouraux and Plaghki, 2006). Indeed, sudden and intense stimuli of other sensory modalities have been demonstrated to elicit transient cortical responses whose main components (the N and P waves maximal at the vertex) are largely similar to those elicited by laser pulses (Mouraux and Iannetti, 2009). The similarity between the response elicited by transient auditory stimuli and by laser nociceptive stimuli (Hu et al., 2015) suggests that this is the case also in rodents. Further investigations of the similarities between the cortical response elicited by stimuli of different modalities, as well as their sensitivity to contextual factors (i.e., stimulus repetition, sensitivity to changes in different stimulus features) are needed to achieve a more comprehensive characterization of the functional significance of murine C-LEPs.

Importantly, even if murine C-LEPs reflect multimodal cortical activity not obligatorily related to nociception, these responses still rely on the functional state of the nociceptive system, both at peripheral and central levels. Indeed, the eliciting afferent input is selectively nociceptive, as demonstrated by the physical features of the stimulus (Sikandar et al., 2013), as well as by the different latency of the response elicited by forepaw and hindpaw stimulation (Fig. 3). Therefore, when short-term habituation is avoided by delivering the laser stimuli at a variable inter-stimulus interval of at least several seconds (Iannetti et al., 2008), murine C-LEPs can still provide an objective, albeit indirect readout of the functional state of the afferent nociceptive system.

## Figures and Tables

**Table 1:** Proportions of behavioural scores (0–4) at different stimulus energies (E1–E5) and stimulation sites (left forepaw, right forepaw, left hindpaw, right hindpaw).

	Left forepaw					Left hindpaw				
E1	E2	E3	E4	E5	E1	E2	E3	E4	E5
‘Score = 0’	94%	20%	0	0	0	96%	22%	0	0	0
‘Score = 1’	5%	12%	2%	1%	0	2%	22%	4%	0	0
‘Score = 2’	1%	7%	2%	0	0	0	9%	6%	0	0
‘Score = 3’	0	27%	13%	3%	0	2%	21%	29%	12%	10%
‘Score = 4’	0	34%	83%	96%	100%	0	28%	61%	88%	90%


	Right forepaw					Right hindpaw				
E1	E2	E3	E4	E5	E1	E2	E3	E4	E5
‘Score = 0’	92%	19%	2%	0	0	97%	24%	3%	0	0
‘Score = 1’	4%	10%	2%	0	0	3%	17%	4%	1%	0
‘Score = 2’	1%	4%	1%	0	0	0	7%	4%	0	0
‘Score = 3’	1%	22%	15%	2%	0	0	31%	26%	17%	5%
‘Score = 4’	2%	45%	80%	98%	100%	0	21%	63%	82%	95%

## Appendix A Supplementary data

Supplementary material. Laser-generated ultrasounds at five different laser energies.

Supplementary audio1 Laser-generated ultrasound at E1 (1 J).

Supplementary audio2 Laser-generated ultrasound at E2 (1.75 J).

Supplementary audio3 Laser-generated ultrasound at E3 (2.5 J).

Supplementary audio4 Laser-generated ultrasound at E4 (3.25 J).

Supplementary audio5. Laser-generated ultrasound at E5 (4 J).
