# Supplementary material for: Laser-evoked cortical responses in freely-moving rats reflect the activation of C-fibre afferent pathways
Source: Neuroimage. 2016 Mar;128:209–17. doi: 10.1016/j.neuroimage.2015.12.042 (PMC4767222; doi:10.1016/j.neuroimage.2015.12.042)
Supplement: Supplementary file 1 — Supplementary material. Laser-generated ultrasounds at five different laser energies. [file mmc1.docx]

**Supplementary materials for “Laser-evoked cortical responses in freely-moving rats reflect the activation of C-fibre afferent pathways”**

To provide an empirical demonstration that laser stimulation generated ultrasounds detectable by the rat auditory system, we performed a recording of the thermoelastic response generated by laser stimulation, using an ultrasound detector (Mini-2 Bat Detector, SUMMIT, Birmingham, UK). The laser parameters were the same used in Experiment 5 of Hu et al., (2015). Five consecutive laser pulses were delivered at each stimulus energy (five energies, i.e., E1-E5, 1-4 J in steps of 0.75 J). Signals (~40-60 kHz) were converted into audible sounds using the ultrasound detector. As expected, the laser-generated ultrasound was graded with the energy of the laser pulse (Fig. S1 and Supplementary Audio Files). This recording explains the graded early “Aδ-ERP” response reported in Figure 1 of Hu et al., (2015), thus providing additional evidence that this early ERP response is consequent to the activation of the rat auditory system (Hu et al., 2015).


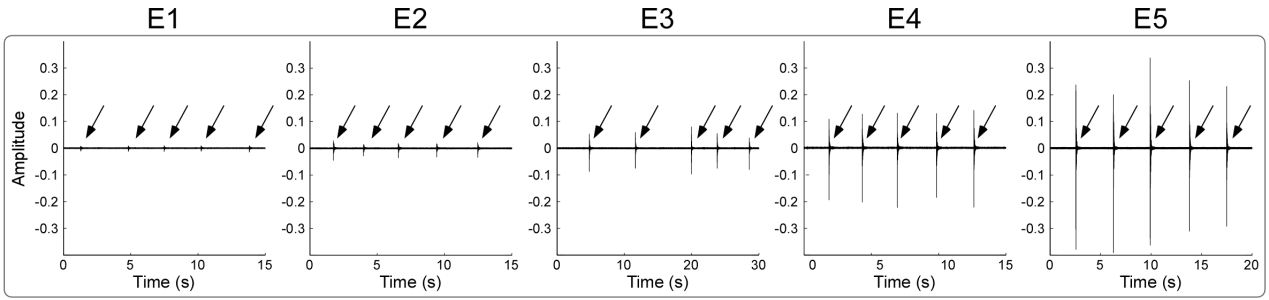


**Figure S1.** Ultrasounds recorded when laser stimuli were delivered at five different laser energies. Five laser pulses (marked with arrows) were delivered at each stimulus energy, Signals (~40-60 kHz) were converted into audible sounds using an ultrasound detector. As expected, the ultrasound consequent to the laser pulse was graded with the energy of laser pulses.

**Reference**

Hu, L., Xia, X.L., Peng, W.W., Su, W.X., Luo, F., Yuan, H., Chen, A.T., Liang, M., Iannetti, G., 2015. Was it a pain or a sound? Across-species variability in sensory sensitivity. Pain 156, 2449-2457.
